# Computational Fluid Dynamics Analysis of Cerebrovascular Hemodynamic Differences in Adults with Sickle Cell Disease: Comparing Stroke and Non-Stroke Cohorts

**DOI:** 10.1007/s10439-026-04112-x

**Published:** 2026-04-08

**Authors:** Lara Abdelmohsen, Anyssa Oden, Tamer S. Ibrahim, Enrico M. Novelli, Sossena Wood, Noelia Grande Gutiérrez

**Affiliations:** 1 Department of Biomedical Engineering, Carnegie Mellon University, 346 Hamerschlag Dr, Pittsburgh, 15213 PA USA; 2 Departments of Bioengineering and Psychiatry, University of Pittsburgh, 3700 O’Hara Street, Pittsburgh, 15260 PA USA; 3 Heart, Lung and Blood Vascular Medicine Institute, University of Pittsburgh, Lothrop St, Pittsburgh, 15213 PA USA; 4 Department of Medicine, University of Pittsburgh, 200 Lothrop St, Pittsburgh, 15213 PA USA; 5 Department of Mechanical Engineering, Carnegie Mellon University, Scaife Hall, Pittsburgh, 15213 PA USA; 6 Neuroscience Institute, Carnegie Mellon University, 4400 Fifth Ave, Pittsburgh, 15213 PA USA; 7 Department of Electrical and Computer Engineering, Carnegie Mellon University, 5000 Fifth Ave, Pittsburgh, 15213 PA USA

**Keywords:** Numerical modeling, Circle of willis, Sickle cell disease, Stroke, Cerebral hemodynamics, Medical imaging-based simulations

## Abstract

**Purpose:**

Sickle cell disease (SCD) is a debilitating genetic disorder affecting hemoglobin in red blood cells. Patients with SCD are at risk of cerebrovascular disease in the Circle of Willis (CoW), with strokes occurring from early childhood into adulthood. Despite this lifelong risk, stroke prevention guidelines using transcranial Doppler ultrasound (TCD) exist only for children, leaving a critical gap for adults. This study aimed to characterize cerebral hemodynamics in the CoW of adults with SCD to support future risk stratification and treatment guidelines.

**Methods:**

Numerical simulations were performed using 3D vascular geometries segmented from high-resolution, patient-specific magnetic resonance imaging in healthy controls (n=3), SCD patients without stroke (n=3), and SCD patients post-stroke (n=3). Key hemodynamic parameters including time-averaged wall shear stress (TAWSS), surface area exposed to low or high WSS, time-averaged mean of maximum velocity (TAMMV), and pressure drop across the CoW were quantified and compared.

**Results:**

Preliminary results show distinct hemodynamic differences were observed across groups. SCD patients post-stroke had lower TAMMV at TCD-equivalent CoW locations (except one with ICA stenosis), the lowest average TAWSS, and the greatest surface area exposed to WSS $<1$ Pa. In contrast, SCD patients without stroke had the highest TAWSS and greatest area exposed to WSS >7 Pa. Despite similar total cerebral blood flow to controls, post-stroke patients showed a lower pressure drop across the CoW.

**Conclusions:**

Patient-specific simulations can quantify cerebral hemodynamics in adults with SCD, offering insight into stroke-related changes and informing future stroke risk assessment and personalized treatment strategies.

## Introduction

Sickle cell disease (SCD) is a debilitating and complex genetic disorder affecting hemoglobin in red blood cells (RBCs), and impacting 100,000 people in the United States and 20 million globally [1]. A single-point mutation causes hemoglobin S to polymerize, making RBCs rigid, adhesive, and sickle-shaped. Cerebrovascular disease, including ischemic strokes, silent infarcts, and hemorrhagic strokes, is a leading cause of morbidity and mortality in SCD and affects patients across their lifespan [2]. Stroke affects 11% of patients with SCD by age 20, 24% by age 45, and recurs in 70% of cases [3, 4]. Further studies report that cerebrovascular disease is the most common neurological complication in 12% of adults with SCD, and 70% show abnormal findings on routine magnetic resonance imaging (MRI) [5, 6]. Despite the high burden of stroke in the aging patients with SCD, there are currently no existing stroke risk stratification or management guidelines for this demographic group [7–9]. The diagnostic and treatment gaps in the management of stroke in adults with SCD stem from a limited understanding of its pathogenesis. The high prevalence of stroke and subsequent cognitive deficits in adults with SCD highlights the need for improved biomarkers to assess stroke risk [10, 11].

The Circle of Willis (CoW) is a common site of cerebrovascular injury in patients with SCD [12]. This ring-like vascular structure distributes blood throughout the brain and frequently exhibits anatomical variations, including hypoplastic or missing segments. Stenosis or occlusion within the CoW, also known as large vessel vasculopathy, has been observed in 31% of patients with SCD and brain tissue damage and is strongly associated with both overt and silent stroke recurrence [13–15]. Histopathological studies in children with SCD and large vessel vasculopathy revealed early intimal hyperplasia in the distal internal carotid (ICA), middle cerebral (MCA), and anterior cerebral arteries (ACA) of the CoW [16–18], although the pathogenesis remains poorly understood. Clinicians currently use non-invasive imaging to monitor the CoW and detect early signs of altered hemodynamics to predict stroke risk in children with SCD. The Stroke Prevention Trial (STOP) established that transcranial Doppler ultrasound (TCD) is effective for identifying children at high stroke risk when blood velocity in the ICA, MCA, or ACA exceeds 200 cm/s [19, 20], and TCD screening is now standard in pediatric stroke prevention guidelines. However, in adults with SCD, TCD is less reliable due to increased skull thickness and the lack of validated velocity thresholds for this population [21], limiting its clinical utility. As an alternative, 4D Flow MRI non-invasively captures blood velocity in all three spatial directions over time and has revealed larger luminal areas, elevated arterial flow, and reduced wall shear stress (WSS) in adults with SCD compared to healthy controls [22, 23]. However, its limited spatial resolution may underestimate WSS, and small adult sample sizes in the studies restrict generalizability. Combining imaging with computational fluid dynamics (CFD) provides a more robust approach for analyzing CoW hemodynamics in adults with SCD by addressing limitations of imaging-only tools such as TCD and 4D Flow MRI. CFD is not constrained by skull thickness and allows for detailed assessment of hemodynamic parameters near the vessel wall. Given the complex pathophysiology of SCD and high inter-patient variability in CoW anatomy, patient-specific CFD modeling is critical for advancing our understanding of cerebrovascular health and improving stroke risk stratification in this population.

Image-based CFD is a powerful tool for simulating patient-specific cerebral hemodynamics, offering high spatial and temporal resolution estimates of velocity, pressure, and WSS, which enable a more detailed understanding of cerebrovascular function and disease progression [24]. CFD has been integrated with MRI and lumped parameter models to develop detailed computational models of the cerebral vasculature [25–27]. These models have been widely used to study cerebrovascular diseases. For instance, studies have quantified flow magnitude and direction through the communicating arteries to assess how CoW anatomical variations influence stroke risk in cardioembolism [28] and demonstrated that higher degrees of ICA stenosis combined with incomplete CoW configurations lead to significant reductions in cerebral perfusion [29]. Other studies modeled collateral activation during vasospasm by simulating directional shifts in flow through the communicating arteries [30]. Pressure and flow obtained from image-based CFD simulations have been proposed as potential biomarkers for cerebrovascular occlusive disease [31, 32], and WSS has been widely used to evaluate arterial wall health, particularly in studies of aneurysm growth and rupture [33–35].

Although CFD has been extensively used in studies of cerebrovascular occlusive disease and cardioembolic stroke, its application to SCD is relatively limited and often does not include a full assessment of the CoW. Early evidence applying CFD in children with SCD reported elevated velocities in the MCA and identified regions of flow disturbance and recirculation [36]. Despite the valuable insights from this early work, only a portion of the CoW was included in the model, which precluded evaluating any compensatory mechanisms to account for local vascular remodeling or anatomical variations. Assuming healthy blood viscosity values is also a limitation in this context since SCD blood viscosity can vary significantly. More recent work has demonstrated the need for patient-specific viscosity and evaluated WSS differences in patients with SCD with and without a history of stroke [37]. Patients with SCD and a history of stroke had higher mean and peak WSS and oscillatory shear indices compared to those without a history of stroke [37]. However, these CFD studies focused on children or young adults, so their findings cannot be extrapolated to older adult populations. The application of CFD on older adults with SCD remains underexplored. Existing studies often rely on generalized boundary conditions, which pose a challenge to making patient-specific predictions. No prior work has examined adults with SCD with stroke history in this particular age range. Our study is the first, to our knowledge, to use patient-specific MRI data to build full-scale CFD models of the complete CoW in adults with SCD transitioning to middle adulthood. We incorporate high-resolution 7T MRI for anatomy reconstruction and use individualized physiological inputs, including blood pressure, heart rate, and hematocrit, to define boundary conditions. We quantify hemodynamic differences that may contribute to cerebrovascular disease, such as velocity, WSS, and pressure variations through the CoW across healthy controls and patients with and without stroke history. This approach fills a critical gap in research for adults with SCD and can contribute to future strategies for stroke risk assessment and management in this understudied population. We hypothesized that hemodynamic compensation is preserved in adults with SCD without stroke but diminished in post-stroke patients, leading to distinct velocity, WSS, and pressure gradients in the CoW.

## Methods

### Patient Demographics

**Fig. 1:**
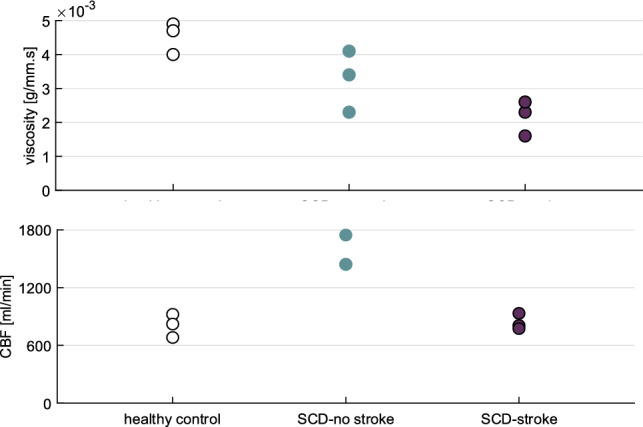
Viscosity and cerebral blood flow (CBF) for healthy controls, patients with SCD without stroke history, and patients with SCD post-stroke

Patients were selected from a previous study that recruited adults with SCD and acquired brain MRI scans to explore neurocognitive markers of cognitive decline in adults with SCD [38]. The study was approved by the University of Pittsburgh Institutional Review Board (IRB PRO12040139), and all participants provided informed consent. The objective of the current study was to investigate hemodynamic differences between patients with SCD with a history of stroke and age and sex-matched patients with no history of stroke and controls. The inclusion criteria for the stroke group were as follows: 1) history of ischemic stroke confirmed by MRI and electronic medical records and 2) age between 18 and 40 years. Exclusion criteria were as follows: 1) vascular malformations such as moyamoya disease and arteriovenous malformations, 2) rare variants of the CoW, such as patients with a trigeminal primitive artery, azygos ACA, and quadruplication of the A2 segment, 3) age below 18 or above 40 years, and 4) MRI scans with motion artifacts, resulting in poorly defined lumens, which prevent the reconstruction of patient-specific vasculature. The age range was specified to exclude subjects older than 40 years to decouple the effects of natural aging on stroke. While the original study included 32 males and 45 female patients with SCD, the patients with history of stroke in this dataset that matched the inclusion criteria were all males (n=3). Thus, age-matched male healthy subjects (n=3) and patients with SCD with no history of stroke (n=3) were selected to match the sex of the post-stroke patients. A total of nine subjects (male=9; age range 27–39) were selected for this study. Hematocrit, blood pressure, and heart rate were extracted from electronic health records for each subject. Details of subject demographics, vitals, stroke history, and genotype are presented in Table 1 and Figure 1. Healthy subjects with HbAA genotype carry two normal hemoglobin A genes. Those with the HbAS genotype carry one normal hemoglobin A gene and one hemoglobin S gene; they are considered carriers (sickle cell trait) and typically do not develop SCD. Individuals with the HbSS genotype inherit two hemoglobin S genes, resulting in the most severe form of SCD. Other forms of SCD arise when a hemoglobin S gene is inherited with another abnormal hemoglobin variant, such as hemoglobin C (HbSC) or beta-thalassemia (HbS$\beta ^{0}$ or HbS$\beta ^{+}$).

**Table 1:** Subject Data Clinical and Hemodynamic Characteristics

Subject	Age	Genotype	Sex	Stroke	Heart	Blood	Hematocrit
					Rate [bpm]	Pressure [mmHg]	[%]
1	30	HbAA	M	No	60	109/56	–
2	37	HbAA	M	No	66	124/71	45.9
3	29	HbAA	M	No	92	153/91	44.9
4	30	HbSB$^+$Thal	M	No	93	119/75	37.6
5	27	HbSS	M	No	66	108/67	27.6
6	39	HbSC	M	No	58	133/88	41.7
7	28	HbSS	M	Yes	71	104/43	28.2
8	36	HbSC	M	Yes	78	113/74	30.6
9	31	HbSB$^0$Thal	M	Yes	85	132/71	19.1

HbAA are healthy controls. HbSS, HbSC, HbS$\beta ^{0}$Thal  or  HbS$\beta ^{+}$Thal are patients with SCD

### MRI Acquisition and Preprocessing

**Fig. 2:**
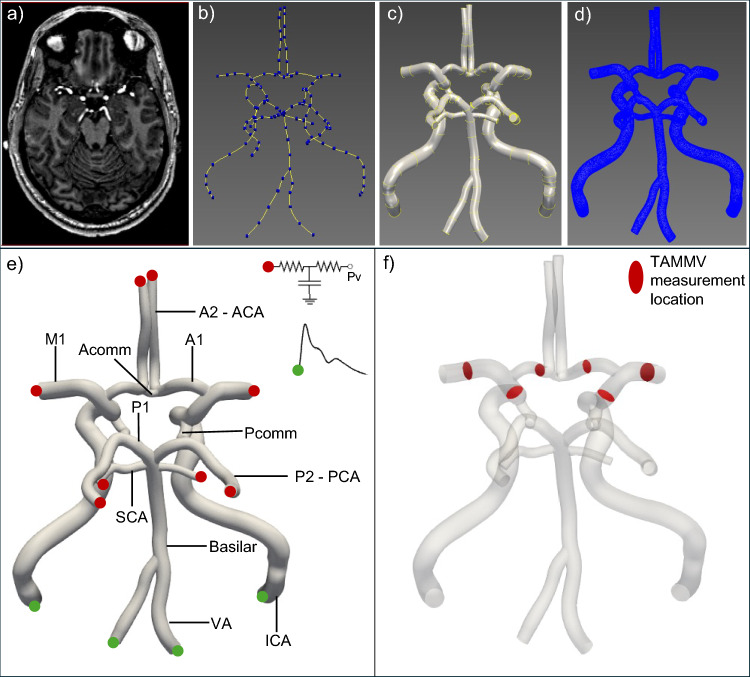
Method for constructing 3D models of the Circle of Willis (CoW) from patient-specific MRI data to run numerical simulations. **a** Patient’s MRI scan. **b** Pathlines tracking the center line of the vessels. **c** 2D segmentations of the vessels and lofted model. **d** Tetrahedral finite element mesh of the model. **e** Outlet boundary conditions represented by an RCR circuit (red) and prescribed flow inlet waveforms on a labeled CoW(green). **f** Locations of measuring time-averaged mean of the maximum velocity (TAMMV) on M1, A1, and ICA segments of CoW

The scans were acquired using a 7T Siemens Magnetom$\circledR$ MRI scanner (Erlangen, Germany) with a customized 1st generation Tic Tac Toe radiofrequency (RF Tac G1) head coil system that has 16-transmit channels and 32-receive channels used in the single transmit mode [39–42]. Anatomical T1 magnetization-prepared rapid gradient echo (MPRAGE) MRI scans were used to visualize blood vessels in the brain. Scans were acquired with 0.75 mm isotropic resolution and TE/TI/TR = 2.17/1200/3000 ms [39]. The scans were bias-corrected and registered using statistical parametric mapping (SPM) unified segmentation algorithm and ConnToolbox (Fig. 2a) [43]. Although time-of-flight scans are the gold standard for visualizing blood vessels, the 7T T1-weighted acquisitions feature more conspicuous blood vessels.

### Arterial Model Reconstruction

Anatomical imaging data from T1 MPRAGE scans were used to reconstruct three-dimensional (3D) geometric models of the intracranial arteries of the CoW in SimVascular, an open-source cardiovascular modeling and simulation software [44]. Individual arteries were tracked on the MRI image to create a centerline path (Fig. 2b). Two-dimensional (2D) segmentations were created using the path lines of the vessels as a guide. The 2D segmentations were lofted to create a 3D solid model of the CoW (Fig. 2c).

**Fig. 3:**
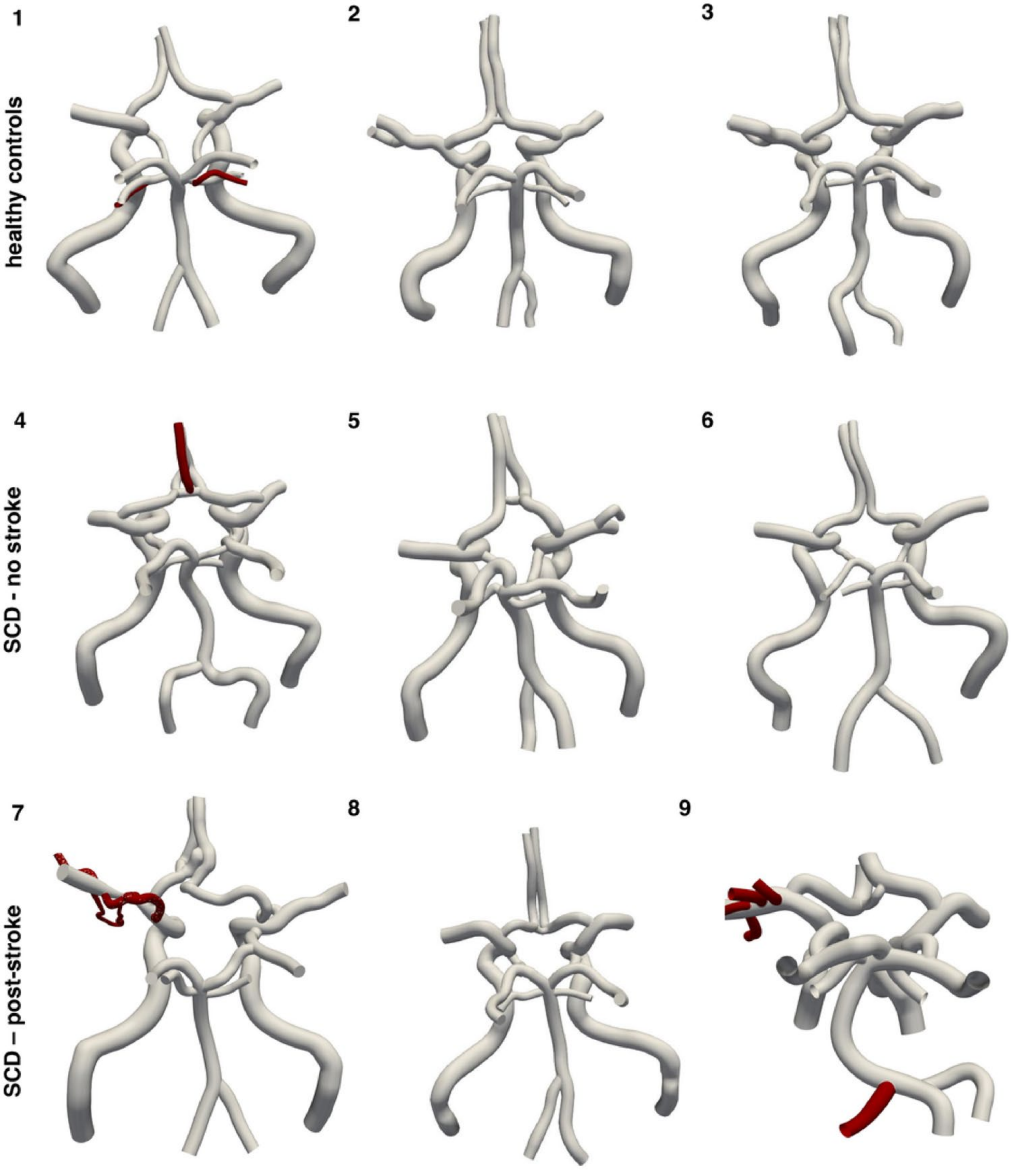
3D vascular models of the Circle of Willis (CoW) of all nine subjects. Vessels in red are patient-specific

The models included the main neck arteries: vertebral arteries (VA) and ICAs, the main intracranial arteries in the CoW: MCAs; M1 segments, ACAs; A1 and A2 segments, anterior communicating artery (Acomm), posterior cerebral arteries (PCA); P1 and P2 segments, basilar artery (BA), posterior communicating arteries (Pcomms), and superior cerebellar arteries (SCAs). Labeled arteries of the CoW on the models can be seen in Figure 2e. Neck vessels were tracked until the C2 vertebra in all models except subject 9, due to lower scan quality leading to limited visibility of the ICAs and VAs. Patient-specific collateral vessels emerging from the CoW were only included if visible in stroke patients. Some subjects had an M1 segment bifurcating earlier than others (for example, subject 2 LMCA in Fig. 3); therefore, this bifurcation was captured in the segmentations instead of a singular M1 branch. Figure 3 shows all nine reconstructed models.

### Numerical Approach

SimVascular’s finite element multiphysics solver [45] was employed to solve the incompressible continuity and Navier–Stokes equations that govern blood flow

1 $$\begin{aligned} \nabla \cdot \textbf{u}&= 0, \end{aligned}$$

2 $$\begin{aligned} \frac{\partial \textbf{u}}{\partial t} + (\textbf{u} \cdot \nabla ) \textbf{u}&= -\frac{1}{\rho } \nabla p + \nu \nabla ^2 \textbf{u}, \end{aligned}$$

where *u* is the 3D velocity field, *p* is the scalar pressure field, $\rho$ is the density, $\nu$ is the kinematic viscosity, and *t* is time. This solver employs a variational multiscale formulation for stabilization, which allows handling transitions to turbulence [46].

For each vascular model, we generated a high-resolution tetrahedral finite element mesh with approximately 3 to 4 million elements and a global edge size of 0.36 mm (Fig. 2d). To capture WSS more accurately, three boundary layers with a gradual 0.5 reduction in edge size were defined. A mesh independence analysis was performed by comparing successive mesh refinements and the addition of boundary layer mesh. Bulk flow metrics, including TAMMV, demonstrated convergence with less than a 3% change across refined meshes. Wall shear stress-based metrics were more sensitive to near-wall resolution and stabilized with the addition of boundary layers, exhibiting less than a 3% change in spatially averaged  time-averaged wall shear stress (TAWSS) and in the surface area exposed to low and high WSS between meshes with two and three boundary layers (Supplementary Fig. 3). A default time step of $10^{-3}$ s was adjusted to each subject’s heart rate for 1000 timesteps per cycle. Although steady simulations are sufficient for computing time-averaged quantities such as TAWSS and the percentage of surface area exposed to low and high TAWSS, transient simulations were required to compute TAMMV. TAMMV is defined as the cycle-averaged peak velocity extracted from time-resolved velocity fields and corresponds to the clinically reported TCD metric used for risk stratification in patients with SCD. Transient CFD simulations ran for three cardiac cycles to reach a converged periodic solution in a high-performance computing cluster. We used data from the final cycle for hemodynamic analysis. Although SCD blood is known to exhibit a pronounced shear-thinning behavior, a sensitivity analysis demonstrated minimal differences between Newtonian and Carreau-Yasuda models at the high shear rates (>100 s$^{-1}$) present in the CoW, supporting the Newtonian fluid assumption used in this study. Therefore, blood was modeled as a Newtonian fluid with a density of 0.00106 g/mm$^3$ and blood viscosity was estimated using the data by Schmalzer et al., which accounts for hematocrit-dependent viscosity under fully oxygenated conditions (PO$_2$=150 mmHg). This choice is appropriate for modeling flow in the CoW, where arterial blood is expected to remain oxygenated [47].

### Boundary Conditions

Subject-specific arterial pressure and heart rate were used to inform inlet and outlet boundary conditions. Flow data was not available from the original dataset to prescribe flow waveforms at the inlets. Instead, we prescribed a reconstructed flow waveform at the four inlets: left and right ICAs and VAs (Fig. 2e). The mean and peak flows in each artery were estimated from a prospective phase-contrast MRI study currently conducted in our lab (Supplementary Fig. 1) that outlined the relationship between vessel cross-sectional area and flow for healthy subjects and adults with SCD, similar to the work by Cebral et al. (2008) [48]. Patients with SCD without stroke tend to be in a state of cerebral hyperemia characterized by increased cerebral blood flow to compensate for their chronic anemia [49, 50]. Thus, separate power law fits were derived for healthy controls and adults with SCD without a history of stroke to capture group-specific differences in cerebral blood flow. These fits were used to estimate inlet flows based on the measured cross-sectional areas at the inlets of the reconstructed 3D model. For adults with SCD post-stroke, inlet flows were estimated using the area–flow relationship of healthy controls, as they are expected to have lower cerebral blood flow than patients with SCD without stroke history [51]. A template flow waveform from an MRI study that characterized volumetric flow rate waveforms in the ICAs and VAs [52] was scaled to match the estimated mean and peak flow for each inlet. All inflow waveforms were phase-aligned as they were derived from the same template waveform. The duration of one cardiac cycle was determined using the subject’s heart rate.

A 3-element Windkessel (RCR) model was used to impose the outlet boundary condition. The total resistance was calculated as the ratio of the arterial-venous pressure drop and total cerebral blood flow. The arterial-venous pressure drop, $\Delta P$, was estimated as the difference between the mean arterial pressure, calculated using the systolic and diastolic blood pressures from the subject’s health record, and the assumed venous pressure of 5 mmHg [53, 54]. The flow for individual inlets based on the power law fit was used to calculate total cerebral blood flow, which was scaled by the area of the outlets for the calculation of resistance at each outlet (Eq. 3). Post-stroke patients were assumed to have a similar blood flow to healthy controls due to an impaired hyperemic response [50]. The total cerebral blood flow estimated for each subject can be seen in Figure 1.

3 $$\begin{aligned} R_{\text {outlet}, i} = \frac{\Delta P}{Q_{\text {total}} \cdot \left( \frac{A_{\text {outlet}, i}}{\sum _j A_{\text {outlet}, j}} \right) } \end{aligned}$$

Proximal resistance was assumed to be 10% of the total distal resistance at each outlet. The capacitance values were assigned based on a previous study that estimated total arterial compliance in the neck and head arteries and proportionally distributed it among vascular outlets (e.g., cerebral, carotid, subclavian arteries) based on their relative contributions to total resistance[31]. The RCR model was tuned until the pulsatile simulations resulted in systolic and diastolic blood pressure in the ICAs within 15% of the subject’s recorded blood pressure and a mean arterial pressure within 10% of the subject’s mean arterial pressure (Supplementary Fig. 2). This deviation from the clinical measurement is justified by the inherent measurement variability of blood pressure cuffs, which can introduce errors in measured values [55]. A no-slip boundary condition was applied at the vessel walls, which were assumed to be rigid. This assumption is valid as intracranial vessels do not show radial deformations that exceed 5% of the vascular diameter [56].

### Analysis

For each simulation, we quantified cerebrovascular hemodynamics in healthy controls and patients with SCD with and without a history of stroke by analyzing key parameters associated with cerebrovascular disease [57]. Specifically, we measured the TAMMV in the M1, A1 segments, and distal ICA, as elevated velocities in these arteries are indicative of potential stenosis and increased stroke risk (Fig. 2f) [20]. In addition to TAMMV,  TAWSS we calculated the spatially-averaged TAWSS and quantified the distribution of TAWSS across the CoW vasculature to identify the range of TAWSS values for each subject. We calculated the interquartile range (IQR) of each subject’s TAWSS histogram to assess the variability in WSS distribution. Group-averaged IQR values were then computed for each cohort. A larger IQR indicated a wider spread of WSS values across the vasculature, reflecting greater spatial heterogeneity in hemodynamic forces. Based on prior literature, we classified WSS values above 7 Pa as supra-physiological and values below 1 Pa as subphysiological, considering that the normal physiological range lies between approximately 2–3 Pa, with lower boundaries reported as low as 1.8 Pa [58]. Low WSS values ($< 0.9\,\text {Pa}$) have been linked to endothelial activation and subsequent arterial remodeling. We also quantified the pressure drop from the inlets to each of the major CoW outlets. For statistical analysis, we used the Kruskal–Wallis test to assess overall group differences, as it is a non-parametric test appropriate for small sample sizes and does not assume normal distribution. When Kruskal–Wallis indicated statistical significance ($p <0.05$), we performed pairwise comparisons using the Mann–Whitney U test.

## Results

### Patients with SCD Without Stroke History Exhibit the Highest TAWSS

**Fig. 4:**
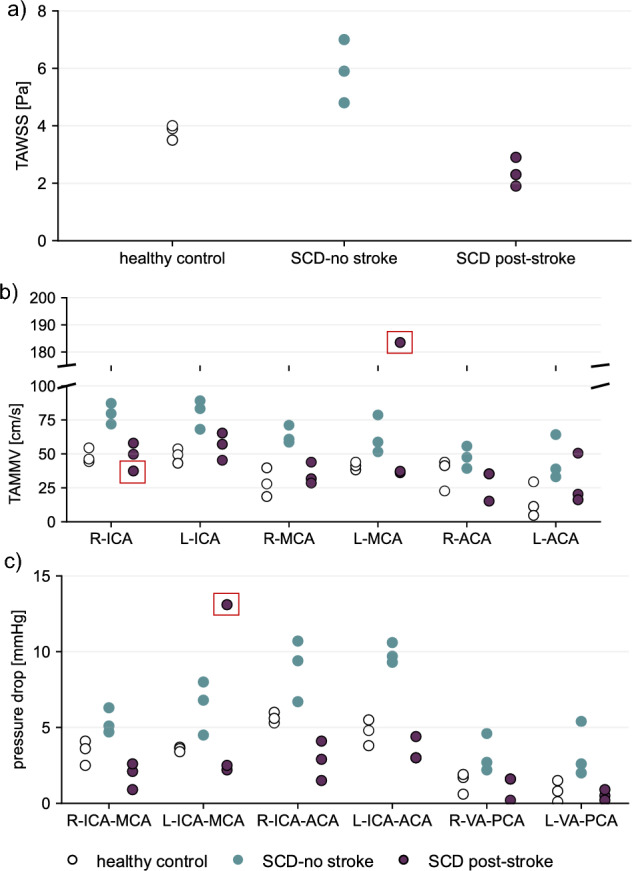
Quantification of hemodynamics in the Circle of Willis. (CoW). **a** Spatial average of TAWSS in the CoW for healthy controls, patients with SCD without stroke history, and patients with SCD post-stroke. **b** TAMMV measured in the MCA, ACA, and ICA of healthy controls, patients with SCD without stroke history, and patients with SCD post-stroke. Red box indicates an outlier due toLMCA stenosis in patient 7 and lowest velocity in RICA in patient 8 measured distal to stenosis. **c** Pressure drop from inlets to the outlets of the CoW for healthy controls, patients with SCD without stroke history, and patients with SCD post-stroke. Red box indicates an outlier due to LMCA stenosis in patient 7

Patients with SCD without stroke history exhibit the highest spatial average TAWSS in the CoW (5.9 ± 0.3 Pa), and patients with SCD post-stroke had the lowest spatial average TAWSS (2.4 ± 0.5 Pa). Patients with SCD post-stroke had TAWSS values approximately 38% lower than healthy controls and approximately 60% lower than patients with SCD without stroke history (Fig. 4a). Patients with SCD and without stroke history had TAWSS values approximately 55% higher than healthy controls. There is a statistically significant difference in TAWSS across the three groups (p = 0.027). However, no statistically significant differences were detected in pairwise comparisons, emphasizing that the findings should be interpreted as preliminary given the small sample size.

### Patients with SCD Without Stroke History Exhibit the Highest TAMMV

**Fig. 5:**
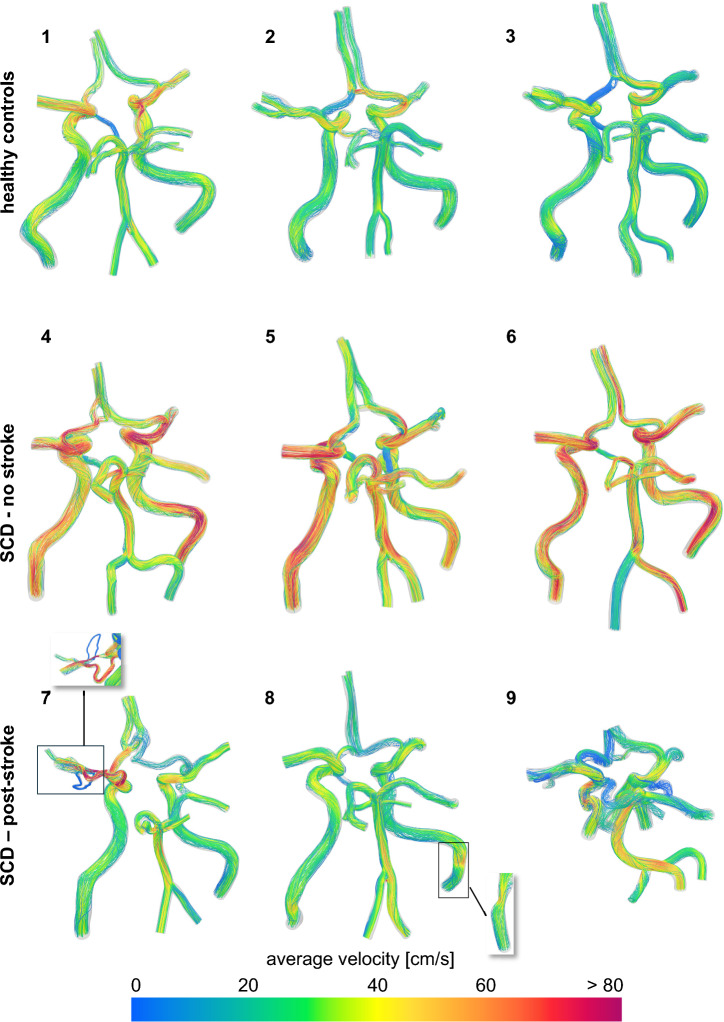
Streamlines in the Circle of Willis (CoW) of all nine subjects. 1-3: healthy controls, 4-6: patients with SCD without stroke history, and 7-9: patients with SCD post-stroke. Boxes show detail on LMCA stenosis in patient 7 and RICA stenosis in patient 8

Velocity measurements, equivalent to TCD velocities in the MCA, ACA, and ICA vessels, were highest in patients with SCD without stroke history (Fig. 4b). Figure 5 shows the average velocity field for all subjects. Increased velocities in red through all vessels can be seen in patients with SCD without stroke. Patients with SCD post-stroke generally had lower velocities than those without a history of stroke. However, LMCA velocity is elevated in one patient in the post-stroke group with a focal stenosis in the LMCA, which increased velocity in the M1 segment. In patient 8 (SCD post-stroke), who has bilateral ICA stenoses, TAMMV measurements were obtained distal to the stenotic segments. Notably, this patient exhibited the lowest TAMMV values in both the right and left ICAs. Our results demonstrate that patients with SCD without a history of stroke exhibit elevated average TAMMV in the ICA, MCA, and ACA compared to healthy controls: 79.9 cm/s vs. 48.5 cm/s (ICA), 63.2 cm/s vs. 34.9 cm/s (MCA), and 46.5 cm/s vs. 25.6 cm/s (ACA), respectively. Patients with SCD post-stroke showed moderately elevated TAMMV compared to healthy controls: 52.1 cm/s vs. 48.5 cm/s (ICA), 60.2 cm/s vs. 34.9 cm/s (MCA), and 28.9 cm/s vs. 25.6 cm/s (ACA), but these elevations were less pronounced than those observed in SCD patients without stroke history. Despite these trends, pairwise group analysis did not show significant differences due to the small sample size. RMCA shows the strongest group difference (p=0.05), especially between controls and patients with SCD without stroke history, and between patients with SCD without stroke history and patients with SCD post-stroke. Other arteries show no significant differences, though RACA trends toward significance (U=9.0, p=0.08).

### Patients with SCD Without Stroke History Have the Highest Pressure drop From Inlets to Outlets

Statistically significant differences in pressure drop exist between groups for RMCA, RACA, and LACA ($p<0.05$). There were no significant group differences in LMCA, RPCA, and LPCA (Fig. 4c). No pairwise comparisons reached $p <0.05$, but there are consistent trends where patients with SCD without stroke history have higher pressure drops than patients with SCD post-stroke, and healthy controls have a lower pressure drop than patients with SCD without stroke history in RMCA, RACA, LACA, and RPCA.

### Patients with SCD Without Stroke History Have a Larger Surface Area Fraction of the CoW Exposed to TAWSS below 1 Pa and above 7 Pa

**Fig. 6:**
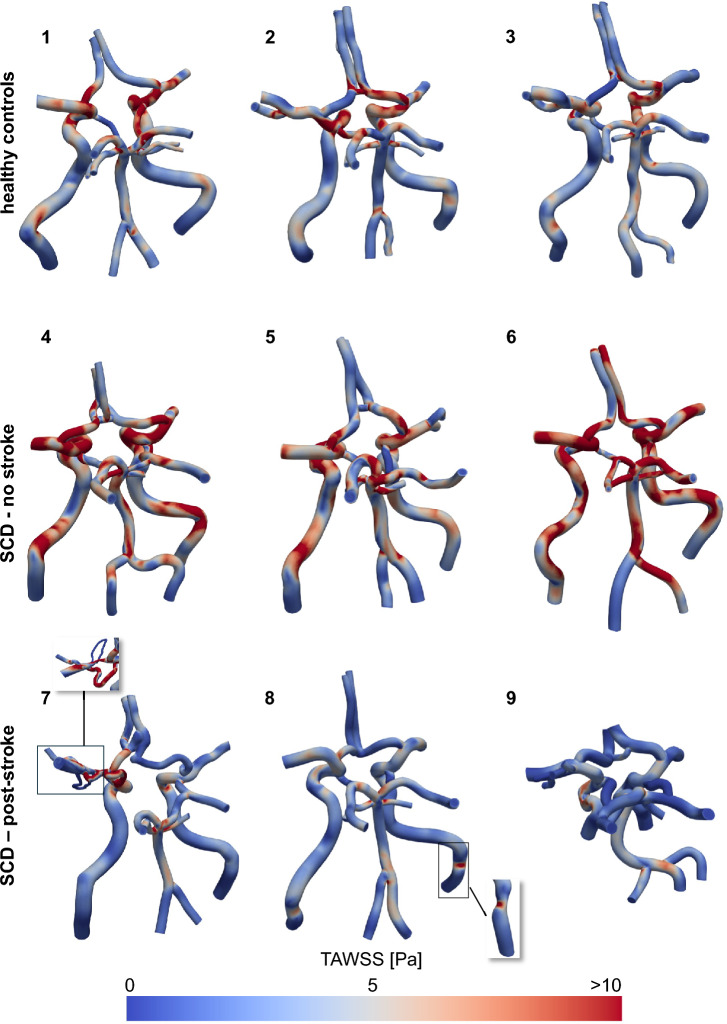
Spatial map of TAWSS in the Circle of Willis (CoW) of all nine subjects. 1-3: healthy controls, 4-6: patients with SCD without stroke history, and 7-9: patients with SCD post-stroke. Boxes show detail on LMCA stenosis in patient 7 and RICA stenosis in patient 8

**Fig. 7:**
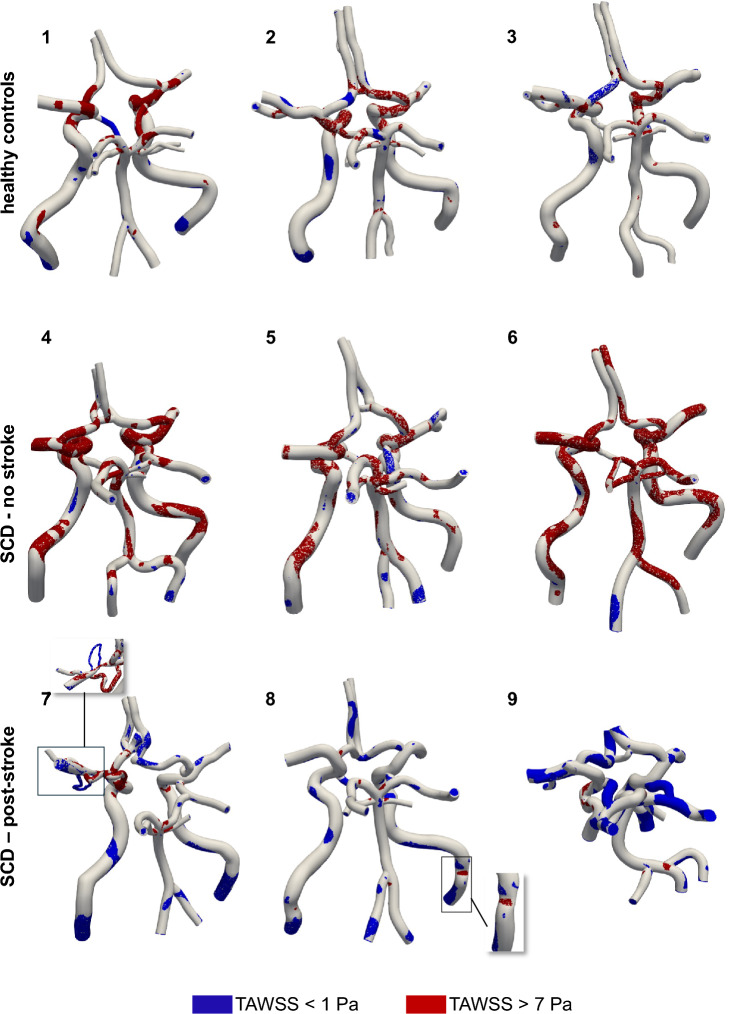
Thresholded spatial maps of TAWSS in the Circle of Willis (CoW) of all nine subjects. Red regions indicate TAWSS above 7 Pa, and blue regions indicate TAWSS below 1 Pa. 1-3: healthy controls, 4-6: patients with SCD without stroke history, and 7-9: patients with SCD post-stroke. Boxes show detail on LMCA stenosis in patient 7 and RICA stenosis in patient 8

**Fig. 8:**
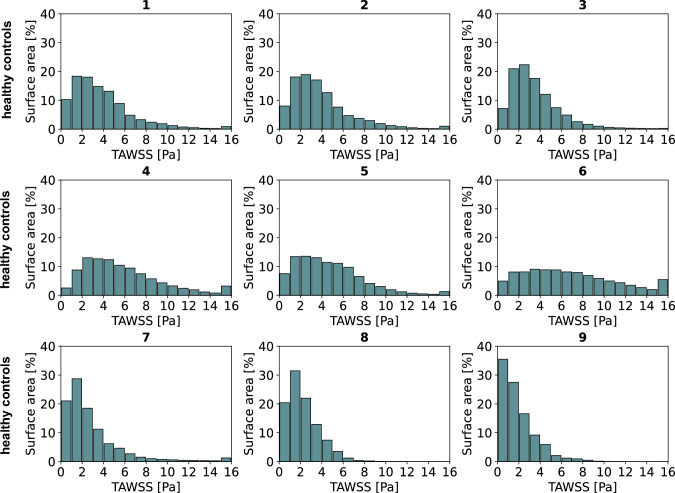
Histograms showing the distribution of TAWSS across the surface area of the Circle of Willis (CoW) of all nine subjects. 1-3: healthy controls, 4-6: patients with SCD without stroke history, and 7-9: patients with SCD post-stroke

Across cohorts, distinct patterns in TAWSS distribution were observed. In healthy controls, less than 11% of the CoW surface area is exposed to TAWSS below 1 Pa or above 7 Pa, with an interquartile range (IQR) of 3.1 Pa, indicating a relatively normal distribution centered within physiological limits (2-3 Pa) (Figs. 6, 7, and 8) [58]. In contrast, patients with SCD without stroke history exhibited a shift toward supra-physiological WSS, with 31.5% of the CoW surface area exposed to TAWSS above 7 Pa (Table 2). The TAWSS distribution was wider and more skewed, as reflected by a larger IQR of 5.1 Pa, suggesting that these patients’ vascular walls are exposed to a wider range of shear stress magnitudes, particularly elevated values. Regions commonly exhibiting high TAWSS included the A1 segment, ICA siphon and petrous segment, and the MCA M1 segment (Figs. 6 and 7). Meanwhile, patients with SCD post-stroke showed a different pattern, with 25.7% of the CoW surface area subjected to TAWSS below 1 Pa. The TAWSS for this group was narrower (IQR = 1.3 Pa) and heavily skewed toward low TAWSS values. Sub-physiological TAWSS areas were primarily localized at the ICA inlet, the inferior curvature of the ICA following the petrous segment, the A2 segment of the ACA, anterior and posterior communicating arteries, collateral vessels, and distal to stenotic regions, whereas the stenosed regions were characterized by elevated TAWSS ($>7\,\text {Pa}$).

**Table 2:** Group-averaged TAWSS metrics derived from histograms

Group	TAWSS [Pa]	IQR [Pa]	% Area $<\,$1 Pa	% Area $>\,$7 Pa
Control	3.8 ± 0.3	3.1	8.5	10.6
SCD no stroke	5.9 ± 1.1	5.1	5.0	31.5
SCD post-stroke	2.4 ± 0.5	1.3	25.7	3.2

## Discussion

Our study is the first to provide a detailed, patient-specific characterization of cerebral hemodynamics in adults with SCD, comparing patients with and without stroke history and sex- and age-matched healthy controls. Using MRI-informed CFD, we quantified TAWSS, velocity, and pressure drop across the CoW. Our models use patient-specific viscosity, and we defined flow inlet waveforms and outlet boundary conditions calibrated using patient-specific blood pressure measurements. This approach offers a practical and reproducible framework for modeling cerebral hemodynamics in SCD using only anatomical MRI, heart rate, and blood pressure measurements, maintaining patient-specificity without relying on advanced flow imaging or invasive measurements. A key advantage of our method is its applicability to SCD patients in this age group, where TCD has limitations and unreliable readings. Similarly, while PC-MRI and 4D Flow MRI are primarily collected for research, our method relies on clinically feasible data sources, as anatomical MRI is routinely obtained for cerebrovascular assessment. Overall, our preliminary findings reveal distinct differences between groups in both global and regional hemodynamics, with important implications for understanding vascular remodeling and stroke risk in SCD. We demonstrate that TAMMV at locations in the CoW equivalent to those where TCD measurements are acquired is lower in patients with SCD post-stroke than in patients with SCD and no history of stroke. There is one exception for a stroke patient who has a stenosis, where the TAMMV is higher than that of patients with SCD and no history of stroke.

We show that patients with SCD post-stroke exhibit the lowest spatially averaged TAWSS in the CoW and the largest surface area fraction of the CoW exposed to TAWSS below 1 Pa. On the other hand, patients with SCD without stroke history had the highest spatially averaged TAWSS and the largest surface area fraction of the CoW exposed to TAWSS above 7 Pa. These observations align with previous findings linking low WSS to endothelial activation and arterial remodeling, key components of SCD-related vascular pathology, and suggest a potential thrombogenic effect of chronically low WSS in these regions [59–62]. In contrast, high WSS, particularly near bifurcations, has been associated with elevated flow impingement and increased risk of aneurysm initiation or rupture in other vascular conditions [63–66]. We also found that patients with SCD post-stroke had a lower pressure drop across the CoW (from inlets to outlets), despite having similar total cerebral blood flow to healthy controls. The presence of stenosis and collateral vessels increased the local resistance and pressure drop in the LMCA in patient 7; however, overall, the pressure drop was lower for the patients with SCD post-stroke. This finding may be explained by the fact that this group also had the lowest blood viscosity, which could reduce resistance and contribute to the lower pressure gradients observed. As a result, patients with SCD post-stroke may remain at increased stroke risk under conditions such as hypotension or increased metabolic demand. Regions with low perfusion reserve are more susceptible to ischemia if baseline pressure gradients are already low.

While CFD has been widely applied to understand cerebral hemodynamics, few studies have explored its application in SCD. We introduced IQRs and TAWSS distribution histograms as a novel approach for quantifying spatial heterogeneity of TAWSS in the CoW. Prior studies have shown that when oscillatory components of WSS are minimal, oscillatory shear index (OSI) values are very low and related metrics such as relative residence time (RRT) become effectively redundant with TAWSS, providing limited additional interpretive value [67, 68]. RRT is more informative in cases of large recirculation regions like those typically found in aneurysms or where transport phenomena are the main focus of the study [69]. Consistent with this rationale, we chose not to report OSI or RRT and instead focused on novel distribution-based measures that represent spatial variability in this anatomy. Our findings build on prior work but provide a richer, individualized profile of cerebral hemodynamics across cohorts. For example, we provide an in-depth analysis not only including the spatially averaged TAWSS but also TAWSS distribution across the CoW, as well as quantifying the extent of the CoW exposed to sub- or supra-physiological TAWSS values to better characterize the hemodynamic profile for each individual. In our cohort, TAWSS in the post-stroke group is lower than in the non-stroke SCD group, scaling proportionally with reduced flow as shown in Figure 1. This trend contrasts with a previous study, which reported higher TAWSS and elevated cerebral blood flow in the post-stroke group compared to the non-stroke SCD group [37]. In our work, the average lumen diameters in the post-stroke group were 3% smaller than those in the non-stroke group. In their study, post-stroke patients had lumen diameters 12% smaller than those without stroke history. These discrepancies likely stem from differences in age, sex, and clinical background. Our adult cohort are 11–14 years older than their study and include healthy controls. Additionally, some of their post-stroke patients received neurosurgical interventions that may have increased cerebral blood flow in the CoW.

Our findings regarding blood flow align with those of a recent 4D Flow MRI study that quantified intracranial hemodynamics in the CoW in a large cohort ranging from 8 to 56 years old [50]. We observed a trend toward increased flow in patients with SCD compared with controls, consistent with their results, and our mean flow velocity values fall within the range reported in that study (Table 3). While time-averaged endothelial shear stress values (or TAWSS) in patients with SCD are similar between the two studies, we observe higher TAWSS values in patients with SCD compared to controls, which differs from their reported trend of lower TAWSS in patients with SCD than controls. This discrepancy may be explained by the differences in velocity and lumen area between controls and patients with SCD in the two studies. From 4D Flow MRI measurements, they report no change in velocity in adults within our age range (controls vs. patients with SCD). However, their study reports larger lumens in adult patients with SCD. Although we do observe larger lumens in patients with SCD, it is possible that the luminal increase is not sufficient to accommodate the increased flow demand without an increase in cerebral blood flow velocity. This would explain the higher velocities observed in the non-stroke group in our study. Since both studies utilize patient-specific viscosity to estimate the shear stress, the elevated TAWSS in our patients with SCD likely results from increased flow occurring without sufficient compensatory dilation. Compensatory remodeling has been observed in *in vivo* studies in SS mice, which have shown expansive luminal remodeling, increased diameter, and altered biomechanical properties [70]. However, the timeline for this remodeling in humans has not been fully characterized. In addition, given the heterogeneity of SCD, remodeling may manifest differently across patients and influence how WSS is distributed along the vessel wall. It is worth noting that in the 4D Flow MRI study referenced above, there were no healthy controls in the same age range that we included in our study, and looking at their data points, it is possible that adding more subjects in this range could change some of the trends observed in WSS. Other studies, like ours, have reported WSS to be higher in patients with SCD than in controls from Doppler flow measurements in the brachial artery, with no significant difference in arterial diameter [71]. Collectively, these contrasting and complementary findings suggest that patient-specific anatomy and flow rate, rather than viscosity alone, play a critical role in determining hemodynamic shear stress. While some studies include a wide age range, a notable limitation is the underrepresentation of adults in middle adulthood and patients with a history of stroke, which we address in our study through targeted inclusion of this subgroup.

**Table 3:** Comparison of segment-averaged velocities measured using 4D flow MRI and simulated mean flow velocities

Artery	4D flow MRI velocity (n=3) [cm/s]	Simulations mean flow velocity (n=3) [cm/s]
RICA	$60.8 \pm 13.9$	$54.1 \pm 1.3$
LICA	$58.3 \pm 7.7$	$55.3 \pm 3.9$
RMCA	$55.9 \pm 2.4$	$40.9 \pm 2.3$
LMCA	$49.9 \pm 10.5$	$43.0 \pm 5.8$
RACA	–	$29.6 \pm 1.3$
LACA	–	$33.2 \pm 14.2$

Reference values for adults with SCD are less well established, but prior studies have reported higher TAMMV in adults with SCD (110.9 cm/s) compared to healthy controls (71.1 cm/s), with the difference proportional to the degree of anemia [72]. Another study using TCD identified a TAMMV threshold of 123.5 cm/s for detecting intracranial MCA or ICA stenosis in adults with SCD [6]. In our cohort, patients with SCD and no history of stroke exhibited higher flow velocities than controls, but did not reach values above 62 cm/s. In contrast, patients with SCD and a history of stroke generally showed lower velocities than their non-stroke counterparts across all arteries, except for one patient (subject 7), who had LMCA stenosis and TAMMV of 183.5 cm/s. This elevated velocity is consistent with the study in adults with SCD, that identified a TAMMV threshold of 123.5 cm/s to detect intracranial MCA or ICA stenosis with 100% sensitivity and 73% specificity [6]. While high velocities are often associated with stenosis, abnormally low velocities may also be clinically significant. Although this has not been explored in adults, one pediatric study described five children with sickle cell anemia who presented with screening TCD velocities $\le$70 cm/s; all went on to develop stroke and were started on chronic transfusion therapy to prevent further events. The primary goal of TCD is to detect focal velocity elevations, which may indicate either arterial stenosis, increased cerebral blood flow (hyperemia) to compensate for chronic anemia, or both [73]. However, a key limitation of TCD is its inability to distinguish between these conditions, as a single velocity measurement cannot differentiate stenosis from hyperemia [51, 74, 75]. This distinction is clinically important, since patients with hyperemia may not benefit from indefinite transfusion therapy to the same extent as those with confirmed stenosis. To address this, the STOP trial investigators proposed that unilateral high velocities are more indicative of focal stenosis, while bilateral high velocities may reflect bilateral stenosis, hyperemia, or both [76, 77]. Our CFD approach allows for spatially resolved analysis of velocity (Fig. 5) and pressure throughout the CoW, enabling the identification of localized flow disturbances, pressure gradients, and patterns consistent with either stenosis or increased flow demand. This level of resolution helps differentiate between pathological narrowing and physiologic compensatory flow, which cannot be achieved with TCD alone.

The group-specific hemodynamic patterns observed in this study can be interpreted in the context of disease-dependent compensatory mechanisms in SCD. In chronic anemia, cerebral oxygen delivery is maintained primarily through vasodilation and increased cerebral blood flow, resulting in elevated velocities under baseline conditions [78]. However, prior work has shown that this compensatory vasodilatory state can limit cerebrovascular reserve, such that additional reductions in arterial pressure or increases in metabolic demand may not be met, predisposing the brain to ischemia [79]. Longitudinal studies further suggest that progressively higher cerebral blood flow is required with age to compensate for declining blood oxygen content in SCD, with potential exhaustion of vasodilatory capacity by mid-adulthood [80]. In this context, the lower velocities, reduced TAWSS, and diminished pressure gradients observed in adults with SCD and a history of stroke are consistent with an altered or exhausted compensatory state, characterized by potentially reduced shear-mediated adaptation and increased exposure to subphysiological WSS. These findings support the hypothesis that cerebral hemodynamic compensation differs between adults with SCD with and without prior stroke, with post-stroke patients exhibiting a reduced capacity to sustain compensatory flow under physiological stress.

Numerical uncertainty in the simulations arises from discretization choices (e.g., mesh resolution), constitutive modeling (e.g., Newtonian versus non-Newtonian blood viscosity), and prescribed boundary conditions, contributing to simulation error. Physiological variability, in contrast, reflects patient-specific differences in vascular geometry and flow conditions, intrinsic to real patient populations rather than sources of simulation error. Based on prior studies quantifying geometric reconstruction uncertainty, the expected error introduced by segmentation-related variability is minimal (<2%) for bulk flow and pressure metrics, but larger for WSS–based quantities. Perturbation-based analyses have shown that geometric variations of 0.25–1.0 mm result in <2% uncertainty in flow rates, <1% uncertainty in spatially averaged pressures, and $\sim$10% uncertainty in WSS magnitude, with higher variability during diastole [81]. Similarly, data-driven uncertainty analyses have reported coefficients of variation, defined as the ratio of the standard deviation to the sample mean, of 0.2–3% for pressure, 1.4–15% for velocity, and 3–20% for TAWSS, indicating comparable relative variability across hemodynamic metrics [82]. Sensitivity analyses further indicate that uncertainty in minimum lumen diameter contributes more strongly to hemodynamic variability than other modeling parameters, including lesion length, blood viscosity, and boundary conditions, across both idealized and patient-specific geometries [83].

In the context of the present study, these findings suggest that geometric reconstruction uncertainty is unlikely to alter observed group-level trends in flow and pressure and is expected to introduce variability on the order of 10–20% in WSS-derived metrics. This variability is likely amplified in diseased anatomies with narrowed lumens, such as stenotic vessels, and therefore, higher uncertainty is expected in the group of SCD patients with a history of stroke. The magnitude of reconstruction uncertainty also depends on the imaging modality and inter-operator variability. A sensitivity analysis on lumen segmentation performed by our group revealed moderate inter-operator variability in reconstructed lumen area (25.0 ± 3.1 mm$^2$, n = 4), which introduces geometric uncertainty that may influence downstream flow and WSS estimates. This corresponds to a coefficient of variation of approximately 12%, which is comparable in magnitude to the relative variability reported in prior inter-observer segmentation studies between two medically trained professionals in a 4D CT–based analysis. They reported a Dice similarity coefficient of 0.75 ± 0.06 ($\sim$ 8% relative variability) in the CoW, reflecting substantial uncertainty in vessel boundary definition [84]. Based on prior geometric uncertainty quantification studies demonstrating that lumen diameter is the dominant contributor to WSS variability, the measured inter-operator area variability in this study is therefore expected to propagate to WSS uncertainty of 10–20%. The slightly higher variability observed in the present study may be attributable, in part, to differences in imaging modality, as most prior studies relied on computed tomography imaging with typical in-plane resolutions of 0.4–0.6 mm, whereas our models were reconstructed from 7T MRI data with a slightly lower spatial resolution of approximately 0.75 mm [84, 85].

In addition to geometric uncertainty, uncertainty in prescribed boundary conditions has been shown to introduce variability in simulated hemodynamic metrics. Prior uncertainty quantification studies examining boundary condition and material property variability reported coefficients of variation of approximately 1–3% for velocity and pressure and 10–20% for WSS-derived metrics across both healthy and diseased vascular anatomies [86]. Similarly, stochastic boundary condition analyses in intracranial and coronary vessel networks reported pressure variability on the order of 6–9% and WSS variability ranging from 5–20% [87].

In the present study, inlet flow conditions were estimated from PC-MRI–based cerebral blood flow and lumen area empirical fits and introduce an estimated 10–20% uncertainty due primarily to lumen segmentation. A 10–20% uncertainty in inlet flow is therefore expected to propagate to modest variability in pressure ($\sim$3%) and TAMMV ($\sim$1%), but to substantially larger variability in TAWSS (10–20%), indicating a stronger dependence of wall shear metrics on inlet conditions. Consistent with observations for geometric uncertainty, these findings suggest that moderate uncertainty in boundary condition specification is unlikely to substantially affect group-level trends in flow and pressure, while primarily contributing to variability in WSS-based quantities.

### Limitations and Future Directions

Several limitations should be acknowledged. MRI resolution affects segmentation accuracy, and PC-MRI-based cerebral blood flow estimation introduces 10–20% variability due to lumen segmentation. Model generation is time-intensive, and although inlet waveforms were scaled using patient-specific parameters, they were not directly measured. Outlet boundary conditions assumed healthy downstream microvasculature, and flow was distributed according to outlet lumen area. However, silent cerebral infarcts in distal vasculature are even more common than stroke, affecting more than 50% of adults with SCD [9, 49] and can significantly affect the perfusion of cerebral vascular territories. Prior studies report that MCA and ACA vascular territories, including the frontal, parietal, and basal ganglia, are more likely to show perfusion deficits, while PCA vascular territories such as the thalamus, temporal lobe, brainstem, cerebellum, and occipital lobe are often less impacted in patients with SCD [18, 88]. Additionally, the small sample size in this study limits statistical power, particularly for pairwise comparisons, and may have reduced the ability to detect statistically significant differences. This highlights the preliminary nature of our findings. A power analysis based on preliminary TAWSS results from this study demonstrated a large effect size (Cohen’s *f* = 4.31), indicating that a sample size of 10 subjects per cohort would be sufficient to achieve 80% power ($\alpha$ = 0.05) to detect group differences using one-way ANOVA in future studies, assuming similar effect sizes.

Another limitation of this study is the estimation of inflow conditions in adults with SCD post-stroke using flow–area relationships derived from healthy controls instead of patients with SCD without stroke history. This assumption was consistent with prior evidence suggesting reduced cerebral blood flow in adults with SCD with a history of stroke [51]. However, data on post-stroke cerebral blood flow in adults with SCD remain limited, and future prospective studies with larger patient cohorts are needed to establish group-specific flow–area relationships for this population.

Additionally, we used data from a previous study to estimate viscosity as a function of hematocrit under oxygenated conditions (PO$_2$ mmHg) with 100% sickled cells, as blood within the CoW is expected to be oxygenated [47]. However, the oxygenation of each subject’s blood is unknown and the study used to estimate blood viscosity did not use whole blood, but used washed RBCs. Blood viscosity in SCD is strongly dependent on hematocrit and oxygenation state. Under oxygenated conditions and at patients’ native hematocrit levels, blood viscosity in patients with SCD is generally lower than in healthy controls, primarily due to chronic anemia [89]. This reduction in viscosity is thought to help preserve microcirculatory flow in SCD by lowering vascular resistance, with additional physiological compensation occurring through reduced oxygen affinity and increased cardiac output [90]. However, when hematocrit is normalized to levels typical of healthy individuals (40–45%), blood viscosity in SCD can exceed that of healthy controls under oxygenated conditions, reflecting the altered rheological properties of irreversibly sickled RBCs. Under deoxygenated conditions, sickling increases blood viscosity across all shear rates, emphasizing the critical role of oxygen tension. Consistent with these observations, other studies have shown that whole blood viscosity in HbSS patients is lower than in healthy controls (HbAA) and in HbSC patients when measured under oxygenated conditions [91]. We compared viscosity estimates under oxygenated and deoxygenated conditions using data from Schmalzer et al. for 100% sickled cells [47]. Our analysis showed that despite differences in viscosity between oxygenated and deoxygenated conditions, and although the absolute WSS values increase with higher viscosity, the trends observed in our study remain unchanged (Supplementary Fig. 4). This demonstrates that the differences observed among groups are driven by patient-specific anatomy and viscosity [47].

Future studies should integrate additional MRI sequences, patient-specific measured inlet flow waveforms, and outlet boundary conditions informed by cerebral perfusion data to improve accuracy. The impact of CoW variants on hemodynamics highlights the importance of a patient-specific hemodynamic assessment. A meta-analysis of 2,718 subjects found that any anatomical variation in the CoW increased ischemic stroke risk by 1.38 times, reinforcing the need to model patient-specific anatomy of the CoW [92]. Moreover, the variability of total cerebral blood flow across individuals emphasizes the importance of measuring patient-specific inflow. While 15–20% of cardiac output ( 750 ml/min) is typically cited as cerebral blood supply, PC-MRI studies have shown a wide range of ICA and BA flows [48, 52]. The hemodynamic impact of CoW variants and high variability in cerebral blood flow across populations highlights the necessity of patient-specific modeling. A full-scale multimodal study combining fMRI-based functional connectivity assessments with cerebrovascular hemodynamic modeling in patients with SCD could yield new insights into how altered perfusion affects pain processing and cognitive deficits in adults with SCD. This approach could help elucidate the role of neurovascular coupling, especially given the suspected link between cortical atrophy, impaired cognition, chronic pain, and regional hypoperfusion in these patients.

Finally, we note that subjects were all male and on different disease-modifying therapies, which may influence findings. Although intracranial aneurysms are reportedly more common in women with homozygous SCD, especially between ages 30–39, silent infarcts are more prevalent in men [93, 94]. Males with SCD have higher risks of cerebrovascular disease, increased inflammation, and a reduced response to hydroxyurea [95]. Some studies suggest that women with SCD have higher vascular reactivity and thus are better able to increase oxygen delivery to the brain under stress, leading to fewer silent infarcts in women [96]. These sex-based differences in cerebral vasculopathy warrant further investigation into how they shape hemodynamics and clinical outcomes in SCD.

### Conclusion

While prior studies have shown that SCD alters cerebral hemodynamics, our findings add important new insights in adults with SCD. This is the first patient-specific CFD study specifically focused on adults with SCD and that quantifies WSS with high spatial and temporal resolution, TAMMV, and pressure drop across variants of the CoW. By classifying patients with SCD by stroke status, we reveal distinct vascular environments that are not evident from global metrics or TCD alone. Our area-weighted TAWSS histograms introduce a metric for capturing spatial heterogeneity in WSS across the CoW, an important feature for understanding localized disease progression and stroke risk in SCD. Together, these contributions establish a clinically feasible, anatomically detailed framework for studying cerebrovascular disease in an understudied adult population that could contribute to early detection and treatment of stroke in patients with SCD.

## Supplementary information

This article has accompanying supplementary files.
